# Characteristics of vestibular migraine, probable vestibular migraine, and recurrent vertigo of childhood in caloric and video head impulse tests

**DOI:** 10.3389/fneur.2022.1050282

**Published:** 2022-11-30

**Authors:** Qin Zhang, Qiong Wu, Jianyong Chen, Xueyan Wang, Yuzhong Zhang, Shuyun Liu, Lu Wang, Jiali Shen, Min Shen, Xinyi Tang, Ling Mei, Xiangping Chen, Yulian Jin, Jun Yang, Qing Zhang

**Affiliations:** ^1^Department of Otolaryngology Head and Neck Surgery, Xinhua Hospital, Shanghai Jiaotong University School of Medicine, Shanghai, China; ^2^Ear Institute, Shanghai Jiaotong University School of Medicine, Shanghai, China; ^3^Shanghai Key Laboratory of Translational Medicine in Ear and Nose Diseases, Shanghai, China; ^4^Department of Otolaryngology Head and Neck Surgery, Affiliated Hospital of Yanbian University, Yanji, Jilin, China; ^5^Department of Otolaryngology Head and Neck Surgery, Second Affiliated Hospital of Xi'an Jiaotong University, Xi'an, Shanxi, China; ^6^Department of Otolaryngology Head and Neck Surgery, The Affiliated Hospital of Southwest Medical University, Luzhou, Sichuan, China

**Keywords:** caloric test, child, symptoms, vestibular migraine, vertigo, video head impulse test

## Abstract

**Objective:**

Vertigo is very common in children, but the specific diagnosis and characteristics are not clear. The main objective of this study was to analyze the characteristics of caloric test (CT) and video head impulse test (vHIT) in vestibular migraine of childhood (VMC), probable vestibular migraine of childhood (PVMC), and recurrent vertigo of childhood (RVC), which can provide a reference value for their clinical diagnosis.

**Methods:**

We selected VMC, PVMC and RVC patients under 18 years of age from the outpatient Department of Otolaryngology–Head and Neck Surgery between May 2021 and August 2022. All patients underwent vestibular function examinations, including eye movement recording CT and vHIT. CT results depended on whether both canal paresis and directional preponderance were under normal limits, and vHIT results depended on the gain values of vestibulo-ocular reflex. The results of both tests were analyzed according to the disease type.

**Results:**

Among the 81 pediatric vertigo patients aged 5–17 years, 44 were females and 37 were males. According to the type of vertigo, 29 patients (25.80%) were diagnosed with VMC, 11 (13.58%) with PVMC, and 41 (50.62%) with RVC. The abnormal rates of the CT in VMC, PVMC, and RVC patients were 24.14%, 36.36%, and 17.07%, respectively. There was no significant difference in the abnormal rates among the three groups (*P* > 0.05). None of the patients showed abnormal vHIT results (all abnormal rates 0.00%). The abnormal CT rates were significantly higher than those of abnormal vHIT rates (*P* < 0.05).

**Conclusions:**

VMC, PVMC, and RVC are more likely to be diagnosed by symptoms, as neither CT nor vHIT are specific to any conditions. Due to different clinical presentations of vertigo in pediatric patients, it is critical to further clarify the diagnosis with medical history and clinical characteristics.

## Introduction

The prevalence of vertigo and dizziness in pediatric patients ranges from 0.4 to 15% (1). In 2021, the Committee for the Classification of Vestibular Disorders of the Bárány Society (ICVD) and the Migraine Classification subgroup of the International Headache Society proposed the diagnostic criteria for vestibular migraine and recurrent vertigo occurring in childhood. For the first time, the new consensus reclassified and formulated the diagnostic criteria for children's vestibular migraine and recurrent vertigo, and defined three disorders: “vestibular migraine of childhood” (VMC), “probable vestibular migraine of childhood” (PVMC), and “recurrent vertigo of childhood” (RVC) (2).

As most children cannot describe their symptoms precisely, it is difficult to distinguish these types of vestibular disorders accurately. The correct diagnostic evaluation of vertigo in children includes not only a detailed medical history and careful physical examination, but also further vestibular tests based on clinical indications (3). The caloric test (CT) and video head impulse test (vHIT) are the most accepted methods for evaluating peripheral vestibular function (4, 5). The CT is a traditional tool to reflect the function of the left and right horizontal semicircular canals (HSCs) and evaluate the status of vestibular function at ultralow frequencies by detecting the vestibulo-ocular reflex (VOR) function in HSCs (6). A new tool, vHIT, comprehensively assesses the function of the three pairs of semicircular canals (HSCs, posterior semicircular canals [PSCs], and anterior semicircular canals [ASC]) at physiological frequencies (7–10). The vHIT and CT are useful in determining the side and severity of affliction in patients with unilateral lesions. However, the two tests might occasionally produce dissociated results in cases of peripheral vestibular disorders (11), such as vestibular neuritis (12, 13) and Meniere's disease (14–16).

Therefore, it remains unclear whether the results of CT and vHIT are dissociated in pediatric patients with vertigo. Thus, in order to improve the accuracy of clinical diagnosis, this article aimed to compare the characteristics of the CT and vHIT in patients with VMC, PVMC, and RVC, and explored the site and possible pathogenesis of vestibular disorders.

## Methods

### Research participants

The study retrospectively reviewed 29 patients (age 10.90 ± 2.94 years) diagnosed as having VMC, 11 patients (age 11.45 ± 3.01 years) diagnosed as having PVMC, and 41 patients (age 9.51 ± 2.61 years) diagnosed as having RVC. Patients were treated in the Diagnosis and Treatment Center of Hearing Impairment and Vertigo, Xinhua Hospital, Shanghai Jiaotong University School of Medicine, Shanghai, China, from May 2021 to August 2022. The vestibular function of all patients was examined by videonystagmography (VNG) examination and vHIT. The specific parameters and response rates of the CT and vHIT results were compared and analyzed among the three groups.

### Eligibility criteria

Patients diagnosed with VMC, PVMC, and RVC were selected according to the diagnostic criteria of the Bárány Society and International Headache Society (2).

We included patients whose medical history data were complete and who could be clearly diagnosed; who were able to cooperate with all the tests; and who had not taken vestibular inhibitors and other drugs within the past 48 h.

We excluded patients with intracranial lesions, hereditary neurological diseases, metabolic diseases, or other systemic diseases; ophthalmic diseases; other peripheral vestibular vertigo diseases, such as Meniere's disease; or external and middle ear diseases.

### VNG examination

The participants underwent VNG (VO425, Interacoustics, Middelfart, Denmark), including the spontaneous nystagmus test, gaze test, random saccade test, smooth pursuit tracking test, optokinetic nystagmus test, positional nystagmus test, and CT.

#### Eye movement recording

The patients wore Frenzel glasses and sat upright with their eyes in the horizontal plane and looked at the target without moving their head. After calibration, tests were performed in the order listed as follows: (1) Spontaneous nystagmus test: the patient was asked to look forward in a dark room without any stimulation for at least 60 s, and the presence of nystagmus was assessed. (2) Gaze test: the patient was asked to focus on the visual target at the center and four eccentric positions for at least 20 s; the absence of nystagmus and the ability to maintain gaze were regarded as normal outcomes. (3) Random saccade test: the patient was asked to track a target that moved randomly within the range of 5–30° in the horizontal direction, with a time interval of 2 s; delay time, peak velocity, and accuracy were calculated by the computer, and saccadic hypometria, saccadic hypermetria, saccadic disorder, and saccadic slowing were considered as abnormal outcomes. (4) Smooth pursuit tracking test was performed by asking the patient to track the visual target moving in the horizontal plane at 0.1–0.5 Hz; eye movement curves were recorded at the same time, and the smooth sine curve with the eye movement curve basically consistent with the visual target curve was considered a normal result. Another parameter evaluated was gain, which was the ratio of eye movement velocity to visual target velocity; it was calculated by the computer, and the normal value was generally not <0.6. (5) Optokinetic nystagmus test: the patient was required to count a series of targets that continued moving at a constant speed of 20 and 40°/s; the direction of nystagmus was opposite to the direction of target movement, and the amplitude and bidirectional amplitude of nystagmus were basically symmetrical. (6) Positional nystagmus test was recorded in the CT position for 40 s, and the presence of nystagmus was assessed.

#### CT and analysis

CT was performed in a dark room using an Air Fix air irrigator (Interacoustics, Middelfart, Denmark) with an airflow of 8 L/min at 50 and 24°C. The patients were placed in the supine position with the head raised at 30° to maintain the horizontal canal in a vertical position. The external auditory canal was irrigated with warm air at 50°C and with cool air at 24°C for caloric testing, respectively. The interval between successive irrigations was 5 min. The sequence used to irrigate the ear canal was right warm (RW), followed by left warm (LW), right cool (RC), and left cool (LC).

The peak caloric response for each irrigation was calculated by averaging the slow-phase velocity (SPV) of a few nystagmus beats that had the highest velocities, which were represented by the symbols RW, LW, RC, and LC. The difference between the caloric responses from the right and left ears was quantified by the percentage of canal paresis (CP), and the CP was calculated as [(RW + RC) – (LW + LC)] / (RW + RC + LW + LC) × 100%. Which can be considered as the caloric weakness when the CP-value is over 25%. Another parameter that was evaluated was directional preponderance (DP). DP was calculated as [(RW + LC) – (LW + RC)] / (RW + RC + LW + LC) × 100%, and which can be considered as the caloric weakness when the DP-value is over 30% (17).

### vHIT and analysis

vHIT was performed using a video oculography device (EyeSeeCam, Interacoustics). The patients were instructed to maintain a sitting position and fixate their gaze on an earth-fixed target in front of both eyes. The target height was flush with the sight line of the patients, and the distance between the patients and target was 1.5 m. The participants were given a video-head impulse tester, which was a special spectacle frame with a built-in camera that tracked pupil movements. The camera of the tester was fixed to the upper-left side of the spectacle frame. For calibration, the patients were asked to keep their heads still and gaze at the alternating laser spot on either side of the target. The patients then provided a slow sinusoidal motion of the head while they kept their gaze fixed on the target.

We conducted a formal test after correct calibration according to the software requirements. An experienced laboratory technician delivered at least 20 brief, abrupt, and unpredictable head impulses per side (10–20° angle, duration 150–200 ms, horizontal semicircular canal impulse peak velocity of 150–300°/s, vertical semicircular canal impulse peak velocity of 100–300°/s). The patients were required to keep gazing at a fixed target in front of them during the head-shaking process.

The reduction in VOR gain was defined as semicircular canals dysfunction. The VOR gain value was automatically calculated as the ratio of eye velocity (°/s) to head velocity (°/s), using the device software. vHIT results were considered abnormal when the VOR value of the HSCs at 60 ms was < 0.8 or the VOR value of the vertical semicircular canals (PSC and ASC) was < 0.7 (18). The normal rate was calculated as normal/total × 100.

### Statistical analysis

All data were analyzed using SPSS v. 26 software (IBM Corp., NY, US). The chi-squared test was used to analysis the response rate of the VNG and vHIT and the abnormality rate of each parameter. Continuous data are presented as the mean ± standard deviation. In the multiple comparisons of parameters, the analysis of variance was adopted when the variance was homogeneous; and the H test was used when the variance was not homogeneous. Graphs were produced using R version 4.2.1 (https://www.r-project.org). It is considered statistical significance when *P* < 0.05.

## Results

### Patient characteristics

All of the inclusive 81 children with vertigo were tested for vestibular function examination. The study population was composed of 37 males and 44 females, with a mean age of 10.27 ± 2.86 years (ranging from 5 to 17 years). According to the type of vertigo, 29 patients (25.80%, 14 males and 15 females) were diagnosed with VMC, 11 patients (13.58%, 4 males and 7 females) were diagnosed with PVMC, and 41 patients (50.62%, 19 males and 22 females) were diagnosed with RVC. The baseline characteristics of patients were generalized in Table 1.

**Table 1:** Baseline data of the three groups.

**Characteristics**	**VMC**	**PVMC**	**RVC**	**Total**
Number of patients, *n* (%)	29 (35.80%)	11 (13.58%)	41 (50.62%)	81 (100.00%)
Sex (Male/Female)	14/15	4/7	19/22	37/44
Age (Mean ± SD), years	10.90 ± 2.94	11.45 ± 3.01	9.51 ± 2.61	10.27 ± 2.86

VMC, vestibular migraine of childhood; PVMC, probable vestibular migraine of childhood; RVC, recurrent vertigo of childhood.

### Comparison of eye movement recording results among the VMC, PVMC, and RVC groups

**Figure 1:**
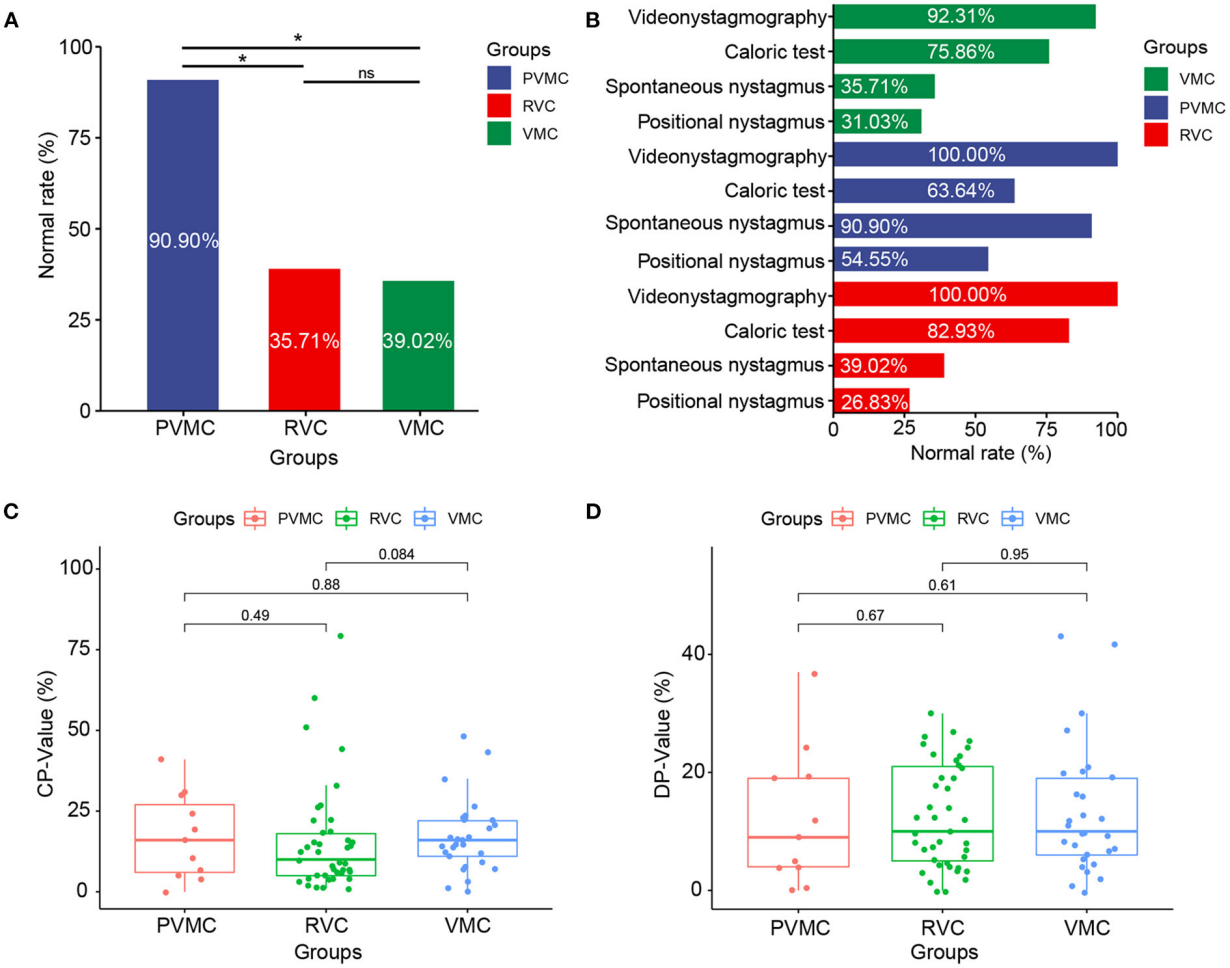
Characteristics of the VNG results. **(A)** Normal rate of spontaneous nystagmus results in patients with VMC, PVMC, and RVC. **(B)** Normal rate of videonystagmography, caloric test, positional nystagmus, spontaneous nystagmus results in patients with VMC, PVMC, and RVC. **(C)** Results of CP-value in caloric tests among the three groups, and **(D)** Results of DP-value in caloric tests among the three groups. *P ≤ 0.05; ns, *P* > 0.05. VMC, vestibular migraine of childhood; PVMC, probable vestibular migraine of childhood; RVC, recurrent vertigo of childhood; CP, canal paresis; DP, directional preponderance.

We calculated the abnormal and normal rates in the total gaze test, random saccade test, smooth pursuit tracking test, and optokinetic nystagmus test as VNG examination response rates among the VMC, PVMC, and RVC groups. During the tests, 3 subjects did not cooperate well with the VNG examination, and at the same time, 1 pediatric patient failed to complete the spontaneous nystagmus test. Almost all of the patients presented with normal results in the VNG examination, while only two (7.69%) patients with VMC showed abnormal VNG results. No statistically significant differences were obtained among the VMC, PVMC, and RVC groups in the VNG examination results (χ2 =3.138, *P* = 0.222; Table 2). Although the majority of participants displayed normal results in the gaze test, random saccade test, smooth pursuit tracking test, and optokinetic nystagmus test, the incidence of positional and spontaneous nystagmus was still high. Eighteen (64.28%) patients with VMC, one (9.09%) with PVMC, and 25 (61.00%) with RVC demonstrated positive results for spontaneous nystagmus. The abnormal rate of spontaneous nystagmus in children with PVMC was lower than that in children with VMC and RVC, and the difference among the three groups was statistically significant (χ2 =10.934, *P* = 0.004) (Figure 1A). Twenty (69.00%) patients with VMC, five (45.45%) with PVMC, and 30 (73.17%) with RVC presented with positional nystagmus in the CT. No statistical difference was calculated in the incidence of positional nystagmus among the three groups (χ2 =3.080, *P* = 0.247; Table 2). The findings of the normal rates for the VNG examination, spontaneous nystagmus test, and positional nystagmus test are intuitively exhibited in Figure 1B.

**Table 2:** Comparison of abnormal VNG rates.

**Test**	**VMC**	**PVMC**	**RVC**	**χ^2^**	***P***
VNG, *n* (%)	2 (7.69%)	0 (0.00%)	0 (0.00%)	3.138	0.222
Spontaneous nystagmus, *n* (%)	18 (64.28%)	1 (9.09%)	25 (61.00%)	10.934	0.004
Positional nystagmus, *n* (%)	20 (69.00%)	5 (45.45%)	30 (73.17%)	3.080	0.247
CT, *n* (%)	7 (24.14%)	4 (36.36%)	7 (17.07%)	1.963	0.374

Fisher's exact test was conducted when the chi-square test conditions were not met.

VMC, vestibular migraine of childhood; PVMC, probable vestibular migraine of childhood; RVC, recurrent vertigo of childhood; VNG, videonystagmography; CT, caloric test; χ^2^, chi-square value/Fisher's exact test value; *P, P*-value.

### Comparison of CT results among the VMC, PVMC, and RVC groups

Among the VMC, PVMC, and RVC groups, the response rate (normal and abnormal) and the specific parameters (CP and DP) of CT were compared. Seven (24.14%) patients with VMC, four (36.36%) with PVMC, and seven (17.07%) with RVC presented with abnormal reactions in the CT. However, there was no statistically significant difference in the abnormal rate of the CT among the three groups (χ^2^ =1.963, *P* = 0.374) (Table 2). Through comparison of specific parameters, we calculated that the CP-values of the CT were 17.28 ± 11.08 in the VMC group, 17.00 ± 13.21 in the PVMC group and 15.54 ± 16.69 in the RVC group. The DP-values of CT were 13.31 ± 11.04 in the VMC group, 12.09 ± 11.58 in the PVMC group, and 12.41 ± 8.80 in the RVC group. Table 3 presents the specific statistical results; no statistical differences were observed among the VMV, PVMC, and RVC groups in either CP-values (*F* = 0.135, *P* = 0.874) or DP-values (*F* = 0.090, *P* = 0.914) (Figures 1C,D).

**Table 3:** Characteristics of nystagmus induced by caloric tests.

**Parameters**	**VMC**	**PVMC**	**RVC**	**F**	**P**
CP (Mean ± SD)	17.28 ± 11.08	17.00 ± 13.21	15.54 ± 16.69	0.135	0.874
DP (Mean ± SD)	13.31 ± 11.04	12.09 ± 11.58	12.41 ± 8.80	0.090	0.914

VMC, vestibular migraine of childhood; PVMC, probable vestibular migraine of childhood; RVC, recurrent vertigo of childhood; CP, canal paresis; DP, directional preponderance; SD, standard deviation.

### Comparison of vHIT results among the VMC, PVMC, and RVC groups

**Figure 2:**
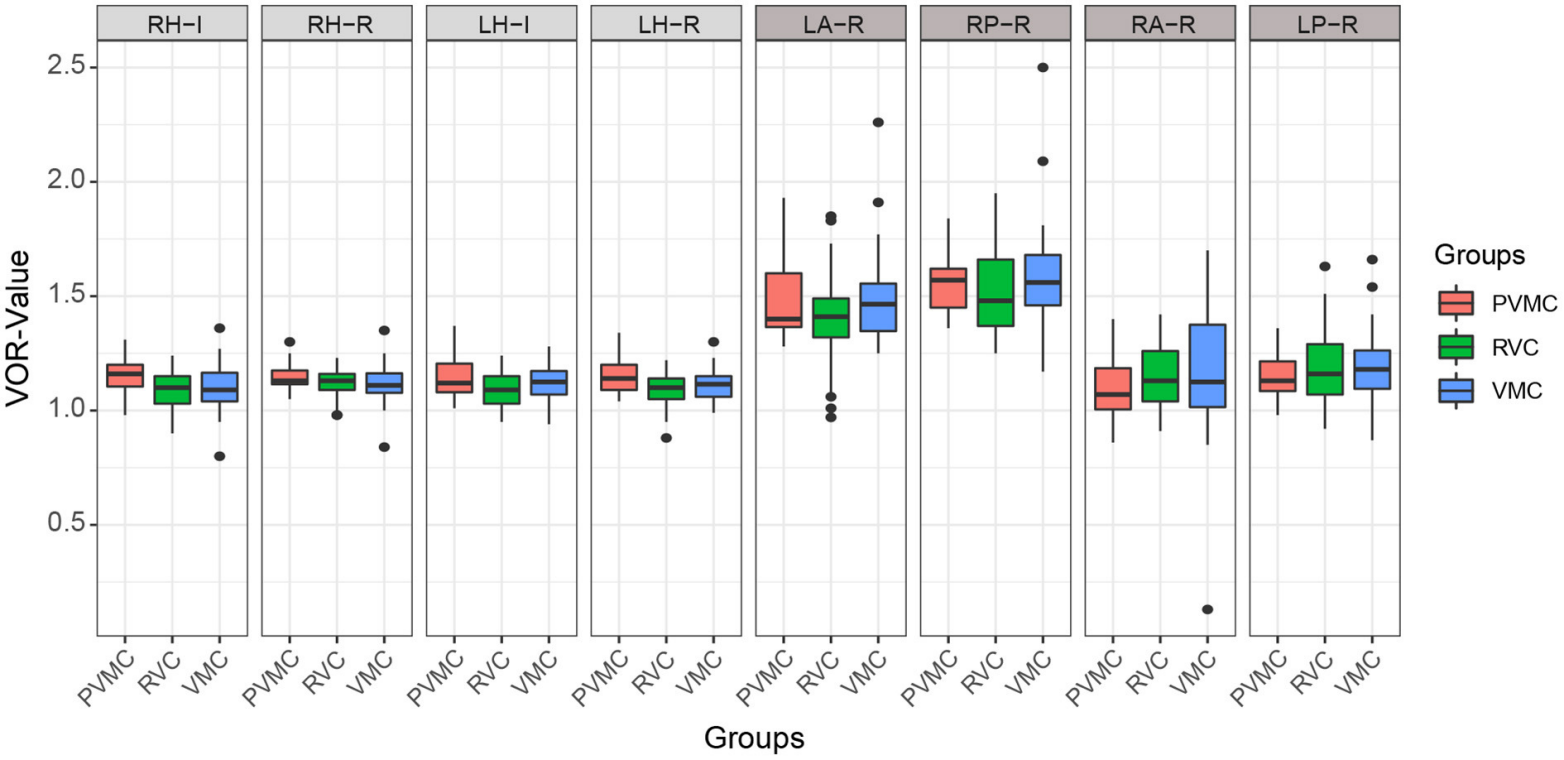
Characteristics of vHIT results. Results of instantaneous and regression VOR gain of each semicircular canals. The center line of the box represents the average VOR gain. RH-I, Instantaneous VOR gains in the right horizontal semi-circular canal; RH-R, Regression VOR gains in the right horizontal semi-circular canal; LH-I, Instantaneous VOR gains in the left horizontal semi-circular canal; LH-R, Regression VOR gains in the left horizontal semi-circular canal; LA-R, Regression VOR gains in the left anterior semi-circular canal; RP-R, Regression VOR gains in the right posterior semi-circular canal; RA-R, Regression VOR gains in the right anterior semi-circular canal; LP-R, Regression VOR gains in the left posterior semi-circular canal.

The instantaneous and regression VOR gain values of the three pairs of semicircular canals are compared in Table 4 and Figure 2. We calculated the instantaneous and regression VOR gain values in the HSCs, including the left HSC (LH) and right HSC (RH), and the regression VOR gain values in the vertical semicircular canals, including the left ASC (LA), right ASC (RA), left PSC (LP), and right PSC (RP). None of the pediatric patients showed an abnormal reaction in the vHIT. There was no statistically significant difference in the vHIT results among the pediatric patients with vertigo (*P* > 0.05).

**Table 4:** Characteristics of VOR gains induced by vHIT.

**Test**	**VMC**	**PVMC**	**RVC**	**F**	***P***
RH–Instantaneous (Mean ± SD)	1.10 ± 0.11	1.15 ± 0.90	1.09 ± 0.09	1.949	0.149
RH–Regression (Mean ± SD)	1.12 ± 0.09	1.15 ± 0.71	1.12 ± 0.07	1.041	0.358
LH–Instantaneous (Mean ± SD)	1.12 ± 0.08	1.15 ± 0.11	1.09 ± 0.08	2.692	0.074
LH–Regression (Mean ± SD)	1.12 ± 0.07	1.15 ± 0.10	1.10 ± 0.07	2.135	0.125
RA–Regression (Mean ± SD)	1.17 ± 0.30	1.09 ± 0.17	1.14 ± 1.45	0.172	0.842
LA–Regression (Mean ± SD)	1.50 ± 0.22	1.49 ± 0.19	1.40 ± 0.20	2.118	0.127
RP–Regression (Mean ± SD)	1.58 ± 0.27	1.57 ± 0.15	1.52 ± 0.20	0.602	0.550
LP–Regression (Mean ± SD)	1.20 ± 0.17	1.15 ± 0.11	1.20 ± 0.16	0.430	0.652

VMC, vestibular migraine of childhood; PVMC, probable vestibular migraine of childhood; RVC, recurrent vertigo of childhood; VOR, Vestibulo-ocular reflex; RH, right horizontal semi-circular canal; LH, left horizontal semi-circular canal; RA, right anterior semi-circular canal; LA, left anterior semi-circular canal; RP, right posterior semi-circular canal; LP, left posterior semi-circular canal.

### Comparison of CT and vHIT results among the VMC, PVMC, and RVC groups

**Figure 3:**
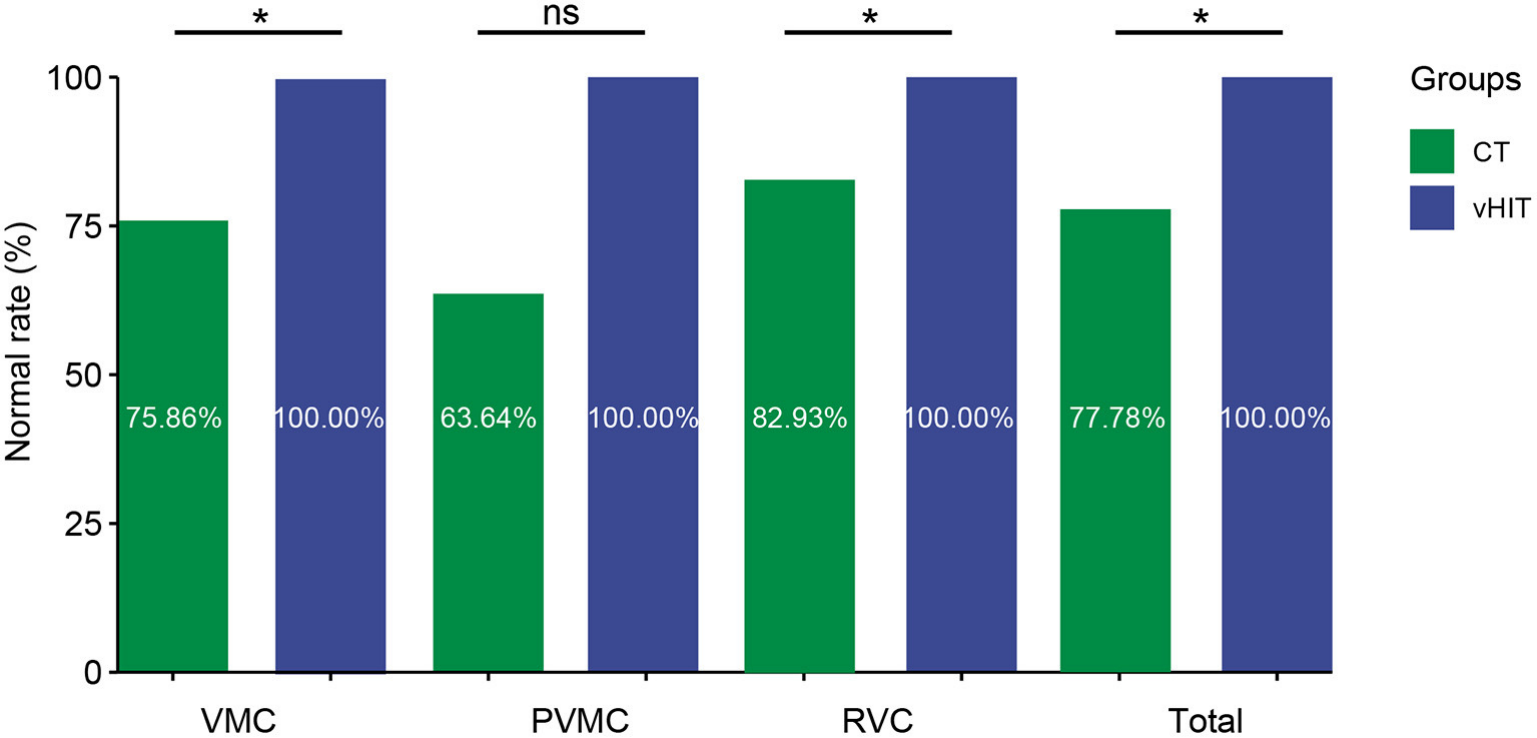
Comparison of normal result rates between CT and vHIT. Comparison of normal result rates between caloric test and vHIT in patients with VMC, PVMC, RVC. *P ≤ 0.05; ns, *P* > 0.05.

We compared and analyzed the results of the CT and vHIT among patients with VMC, PVMC, and RVC. As shown, the abnormality rate in the vHIT among all patients (0.00%) was lower than that in the CT (22.22%) (*P* < 0.001) (Figure 3 and Table 5). The differences between the results of the CT and vHIT in patients with VMC (*P* = 0.01) and RVC (*P* = 0.018) were both statistically significant, whereas no statistical difference was observed in patients with PVMC (*P* = 0.09).

**Table 5:** Comparison of abnormal result rates between CT and vHIT.

**Group**	**CT**	**vHIT**	**χ^2^**	***P***
VMC	7 (24.14%)	0 (0.00%)	Fisher	0.010
PVMC	4 (36.36%)	0 (0.00%)	Fisher	0.090
RVC	7 (17.07%)	0 (0.00%)	5.623	0.018
Total	18 (22.22%)	0 (0.00%)	20.250	<0.001

Fisher's exact test was conducted when the chi-square test conditions were not met.

VMC, vestibular migraine of childhood; PVMC, probable vestibular migraine of childhood; RVC, recurrent vertigo of childhood; CT, caloric test; vHIT, video head impulse test.

## Discussion

As a special group, vertigo in children has challenged clinicians over the years. The diagnosis of vertigo in pediatric patients is difficult, due to the inability of children to describe their symptoms and lack of cooperation with vestibular function examinations. Short-lived manifestations due to rapid compensation make the condition difficult to detect for the parents (19). The most common causes of vertigo and dizziness in childhood are vestibular migraine and benign paroxysmal vertigo, although the frequencies vary among studies (20–22). A consensus on the diagnostic criteria for vestibular migraine and recurrent vertigo in children was proposed in 2021 by the Committee of the International Classification of Vestibular Diseases (ICVD) of the Bárány Society and the Migraine Classification subgroup of the International Headache Society. Their consensus statement updated the diagnostic term benign paroxysmal vertigo to vestibular migraine and recurrent vertigo in children, and reclassified and established the diagnostic criteria for vestibular migraine and recurrent vertigo in children.

The audio vestibular system in humans is anatomically developed and functionally responsive at birth (23). However, only few reports have systematically reported findings on the vestibular function in pediatric patients with vertigo. With the progress and development of medicine, innovation in testing technology makes it possible to evaluate the vestibular function of infants and children with vertigo. CT and vHIT are common diagnostic tools used to evaluate semicircular canal function in order to identify and diagnose vertigo in suspected pediatric patients (24, 25). Thus, we sought to analyze the characteristics of the CT and vHIT in VMC, PVMC, and RVC, with a view to provide a reference for their clinical treatment and diagnosis.

### Comparison of eye movement recording among the VMC, PVMC, and RVC groups

The occurrence of nystagmus is closely related to vertigo. It refers to the patient's inability to continuously focus on the target; the eye moves slowly to one side and deviates from the target, followed by rapid corrective rebound, which can be physiological or pathological. There are three main types of pathological nystagmus: spontaneous nystagmus, staring nystagmus, and positional nystagmus, which are common in peripheral and central vestibular system lesions. In our study, we found that the three groups of patients had spontaneous nystagmus and positional nystagmus of different frequencies.

Spontaneous nystagmus refers to a continuous, involuntary, rhythmic back and forth movement of the eyeball without inducing factors, which can occur in vestibular peripheral lesions, central lesions, and some eye diseases. It is reported that the frequency of spontaneous nystagmus in children with vertigo is ~17–75% (26–31), which is similar to our research results. We found that the incidence of spontaneous nystagmus in the PVMC group was significantly lower than that in the other two groups (*P* < 0.05). However, this may be due to the fact that there were fewer cases in the PVMC group, and the statistical results were not accurate. It was also possible that a patient with PVMC was a possible patient of vestibular migraine, and the clinical symptoms were not obvious, similar to a transitional disease; thus, the extraction rate of nystagmus was not high.

Positional nystagmus refers to the nystagmus that occurs when the head is placed in one or several specific positions, but it does not occur in other positions. It is also accompanied by vertigo, which is called positional vertigo. The mechanism of positional nystagmus is unknown. It is generally believed that it is caused by otolith lesions, but there may also be semicircular canal, vestibular nerve, cerebellum, brain stem, brain, and other lesions. Previous studies found positional nystagmus in only 20% of children with vertigo (32, 33). In our case, we found that the extraction rate of positional nystagmus was significantly high: 69.00% in the VMC group, 45.45% in the PVMC group, and 73.17% in the RVC group. The reason for the high frequency of positional nystagmus may be because the patients we included were children with vertigo who were selected according to the latest diagnostic criteria for children's vestibular migraine and recurrent vertigo, and they had a higher degree of cooperation with the vestibular function test, while previous research focused on vestibular diseases in children, which was more extensive.

### Comparison of CT results among the VMC, PVMC, and RVC groups

The CT belongs to the ultra-low frequency testing method (0.002–0.004 Hz), which mainly detects the function of the supravestibular nerve and horizontal semicircular canals. It can stimulate the horizontal semicircular canal on both sides and is the gold standard for evaluating vestibular function (6). We found no statistically significantly abnormal CT rates among patients with VMC (24.14%), PVMC (36.36%), and RVC (17.07%). Langhagen et al. (34) found that the rate of abnormal CT findings was 21.0 and 25.0%, respectively, in the RVC and VMC patients, and Marcelli et al. (35) conducted a CT on pediatric patients with vestibular migraine and reported an abnormal rate of 33.0%. These findings are similar to our research results, indicating that children with vertigo may have damaged to a certain extent the function of the low-frequency horizontal semicircular canals.

### Comparison of vHIT results among the VMC, PVMC, and RVC groups

The vHIT has been shown to be effective for diagnosing VOR deficits resulting from peripheral vestibular impairment, and allows rapid evaluation of the high-frequency (2–5 Hz) function of the three pairs of semicircular canals (36, 37). Our statistical results showed that none of the pediatric patients had an abnormal reaction to vHIT. Similar results also appeared in the study of Salmito et al. (38), who found that the vestibular function of patients with vestibular migraine was generally normal in the non-acute phase. Additionally, Chen et al. (39) studied 36 RVC patients, and found no statistically significant difference in the abnormal rate of vHIT between the normal control group and the RVC group. This may be because, in daily life, pediatric patients with vertigo receive more high-frequency stimulation to the vestibule, and high-frequency vestibular function is prone to compensation, which leads to a low rate of abnormal vHIT results. Therefore, the vHIT may be used as the main assessment method for the early or decompensated stage of vertigo in pediatric patients. Undoubtedly, the vHIT plays an important role during bedside physical examinations of patients with acute dizziness.

### Comparison of the vHIT and CT results among the VMC, PVMC, and RVC groups

Before the widespread application of vHIT to evaluate vestibular function, the CT was considered as the gold standard of unilateral horizontal semicircular canal dysfunction. However, currently, we can detect abnormalities for any semicircular canal and clearly view overt and covert saccades by vHIT. However, these two tests might occasionally produce conflicting results. Many investigations have verified the dissociation between the outcomes of the two tests, and it is common to obtain a normal result in one of the two tests, while an abnormal result in the other, particularly in peripheral vestibular disorders, such as Meniere's disease (14–16) and vestibular neuritis (12, 13). In our study, we found that 22.2% of pediatric patients showed abnormal CT results, while no abnormal vHIT results were found, which may indicate that the CT was ultimately more sensitive for diagnosing vestibular diseases. Moreover, although both tests estimate the function of horizontal semicircular canals, they have noteworthy limitations and differences. As in cochlear disease, the involvement of the semicircular canals can lead to specific frequency dysfunctions. Due to the limitation of external auditory canal and eardrum, non-physiological stimulation (~0.003 Hz) is required during the CT. Further, CT can only assess the low frequency functional state of the horizontal semicircular canals, whereas vHIT can assess the function of vestibular system under high-frequency (2–5 Hz) stimulation that cannot be determined by CT. The subjects received physiological stimulation similar to head rotation, which will not cause obvious discomfort during the examination. The vHIT may be a useful addition to the existing vestibular function test, however, it does not appear to be an alternative. These tests evaluate different segments and frequency of the semicircular canals, and consequently, it has been proven that the combination of CT and vHIT can more systematically evaluate vestibular function (40–45).

## Limitations

The article detailedly studied the differences between eye movement recording, CT and vHIT-test results among patients with VMC, PVMC, and RVC. It was preliminarily concluded that CT can detect vestibular function more sensitively than vHIT in children with vertigo. Additionally, through the examination of nystagmus, we obtained a better understanding of vertigo in children. Nonetheless, the sample size of the study is still insufficient. In the future, we can analyze the specific characteristics of different nystagmus, and preliminarily identify that nystagmus is caused by peripheral or central diseases. And, future studies need to expand the sample size for prospective verification and include other vestibular function tests to comprehensively assess the characteristics of vertigo in children.

## Conclusion

Comprehensive ontological and neurological examinations, including history, physical examination, audiological assessment, vestibular function test, and imaging studies, are required for the proper diagnosis of vertigo in children. The CT and vHIT showed no specific diagnostic value in our patients with VMC, PVMC, and RVC. Therefore, history taking is of the utmost importance, not only in regard to the various symptoms or observations, but also in regard to the sequencing of symptoms and their progression or lessening over time, as RVC, VMC, and PVMC are more likely to be diagnosed based on symptoms.

## Data availability statement

The original contributions presented in the study are included in the article/supplementary material, further inquiries can be directed to the corresponding authors.

## Ethics statement

Written informed consent was obtained from the minor(s)' legal guardian/next of kin for the publication of any potentially identifiable images or data included in this article.

## Author contributions

QinZ and QW designed the study, collected patients, statistical analyses, and drafted the manuscript. JC, XW, and YZ assisted in drafting the protocol, conducting data collection and processing, and editing the manuscript. SL, LM, JS, and MS performed data extraction. XT, LM, and XC prepared figures and revised the manuscript. QingZ, YJ, and JY critically evaluated the manuscript. All authors reviewed and approved the final version of the manuscript. All authors contributed to the article and approved the submitted version.

## Funding

This research was funded by the National Natural Science Foundation of China (Grant Nos. 82171137, 81970891, and 81860189), the Clinical Research Plan of SHDC (SHDC2022CRD013), the Key International Cooperation Project of Shaanxi Province (Grant No. 2020KWZ-019), and the Science and Technology Project of Shanghai Science and Technology Commission (Grant No. 21S31900600).

## Conflict of interest

The authors declare that the research was conducted in the absence of any commercial or financial relationships that could be construed as a potential conflict of interest.

## Publisher's note

All claims expressed in this article are solely those of the authors and do not necessarily represent those of their affiliated organizations, or those of the publisher, the editors and the reviewers. Any product that may be evaluated in this article, or claim that may be made by its manufacturer, is not guaranteed or endorsed by the publisher.
